# Ampicillin-Sulbactam for Treatment of Carbapenem-Resistant Acinetobacter baumannii Meningitis

**DOI:** 10.7759/cureus.97017

**Published:** 2025-11-16

**Authors:** Shahanas Shajahan, Anup Warrier, Arun Wilson, R Sneha, Akhi Mohanachandran Pushpaleela

**Affiliations:** 1 Internal Medicine, Aster Medcity, Kochi, IND; 2 Infectious Disease, Aster Medcity, Kochi, IND; 3 Infectious Diseases, Aster Medcity, Kochi, IND; 4 Acute Medicine, Royal Infirmary Edinburgh, Edinburgh, GBR

**Keywords:** acinetobacter baumannii, ampicillin-sulbactam, carbapenem resistance, neurosurgical infection, postoperative meningitis

## Abstract

Carbapenem-resistant *Acinetobacter baumannii* (CRAB) is a rare but potentially fatal cause of post-neurosurgical meningitis, primarily due to its broad antimicrobial resistance and limited therapeutic options. We report the case of an eight-year-old girl who developed CRAB meningitis following ventriculoperitoneal shunt surgery. The organism was identified using standard biochemical methods and confirmed with an automated system (VITEK 2 Compact, BioMérieux SA, Marcy-l'Étoile, France). Antibiotic susceptibility testing revealed resistance to carbapenems and susceptibility only to colistin. The patient was treated successfully with a combination of high-dose intravenous ampicillin-sulbactam and intraventricular colistin following shunt exteriorization. Clinical improvement was observed within one week, and cerebrospinal fluid cultures subsequently became sterile. This case highlights the potential role of ampicillin-sulbactam, in combination with colistin, as an effective therapeutic option in managing post-neurosurgical CRAB meningitis, especially in pediatric patients where alternative agents are limited.

## Introduction

Antimicrobial resistance (AMR) is one of the greatest threats to modern healthcare, with substantial clinical, economic, and security consequences. As bacteria develop resistance to even last-line agents such as carbapenems, treating serious infections has become increasingly difficult. The World Health Organization lists carbapenem-resistant *Acinetobacter baumannii* (CRAB) among its highest-priority pathogens [1,2].

*A. baumannii* is an opportunistic gram-negative bacillus that frequently causes hospital-acquired infections such as ventilator-associated pneumonia, bloodstream infections, and post-neurosurgical meningitis. The emergence of CRAB has made the management of these infections particularly challenging due to the organism’s extensive drug resistance and limited therapeutic options [1,2].

In this report, we describe the use of ampicillin-sulbactam to treat post-neurosurgical meningitis caused by CRAB in an eight-year-old child, guided by recommendations from the Infectious Diseases Society of America (IDSA) [1]. The Indian Council of Medical Research (ICMR) has similarly highlighted the declining susceptibility of *A. baumannii* to commonly used antibiotics in India, underscoring the urgent need for effective alternatives [2]. Written informed consent for publication of this case and associated images was obtained from the patient’s legal guardian.

## Case presentation

**Figure 1:**
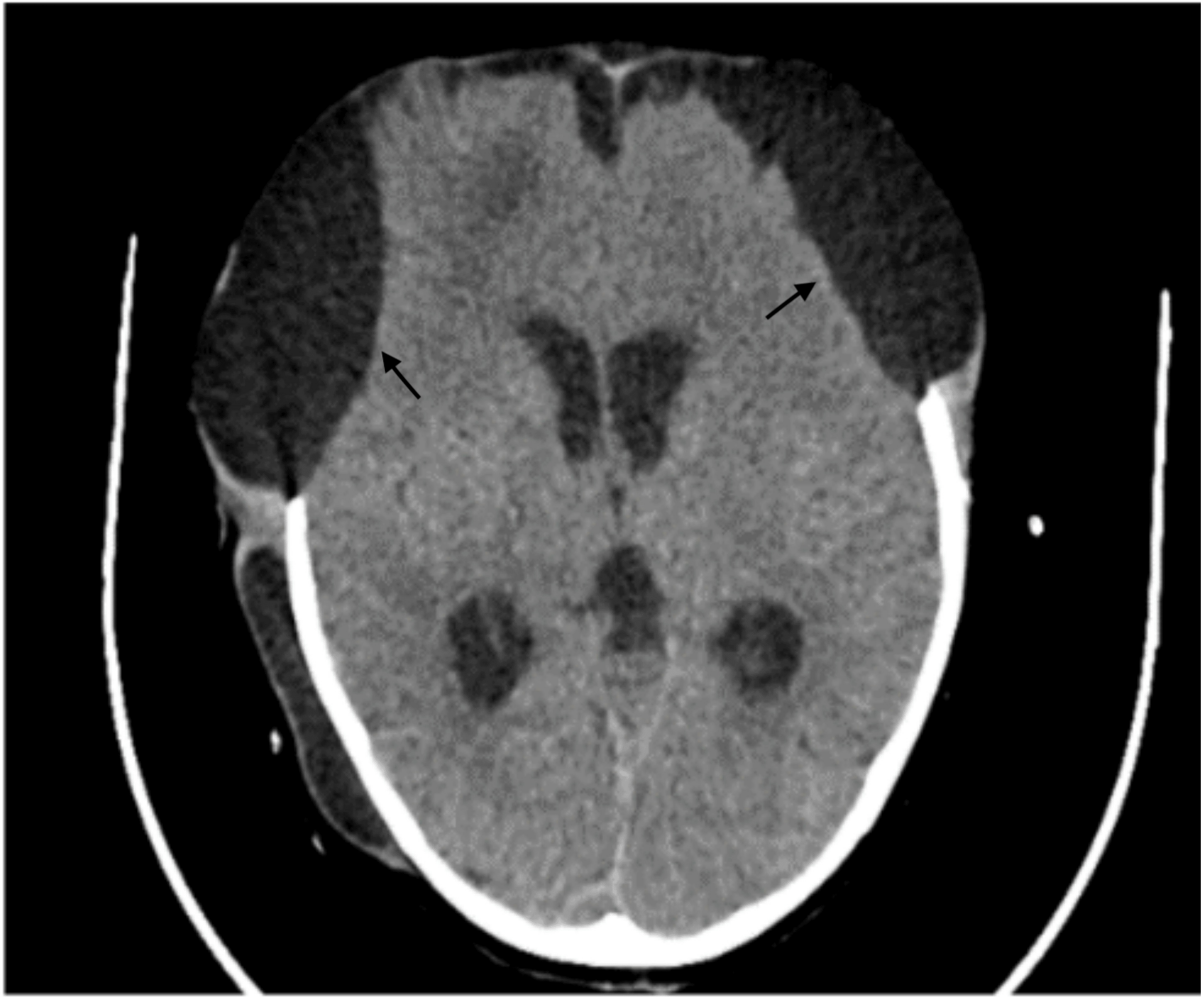
Postoperative MRI shows bifrontal decompressive craniectomy with lax duroplasty and insertion of an external ventricular drain (EVD).

An eight-year-old girl presented to our hospital with a reported history of a road traffic accident and sustained head injury with cerebrospinal fluid (CSF) otorrhea. CT brain revealed subdural haemorrhage encompassing the right temporo-fronto-parietal region, with diffuse subarachnoid and intraparenchymal haemorrhage. She underwent bifrontal decompressive craniectomy with lax duroplasty, and an external ventricular drain (EVD) was inserted for the same (Figure 1).

**Figure 2:**
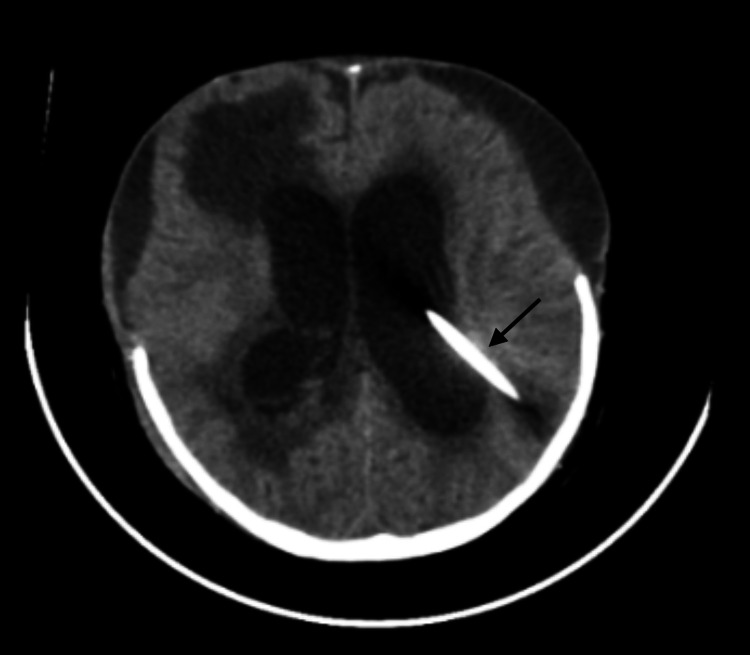
MRI showing VP shunting was done following which she developed CSF otorrhea in the postoperative period.

Following surgery, she developed hypoxia and was maintained on ventilator support. Four days later, she developed ventilator-associated pneumonia, where *Staphylococcus aureus* was isolated as the causative agent, and was treated with cefazolin. After stabilization and after repeat cultures from multiple sites, including CSF, became sterile, ventriculoperitoneal (VP) shunting was performed. In the postoperative period, five days after surgery, she again developed CSF otorrhea (Figure 2), high-grade continuous fever, and multiple seizure-like episodes. There were no radiological or clinical signs of pneumonia at this stage.

A repeat CT revealed increased hydrocephalus with periventricular hypodensity. CSF analysis showed findings consistent with meningitis, including leucocytosis and low glucose. CSF cultures yielded growth of CRAB, which was susceptible only to colistin. The organism was identified using the automated VITEK microbiology system (VITEK 2 Compact, BioMérieux SA, Marcy-l'Étoile, France), along with Gram staining and catalase positivity. Other cultures, including blood and urine, were sterile.

The patient was started on systemic therapy with high-dose ampicillin-sulbactam (three grams thrice daily, based on her body weight of 25 kg) along with intraventricular colistin administered via the EVD following shunt exteriorization. Fever resolution occurred after one week of initiating therapy, with subsequent CSF cultures becoming sterile. Antimicrobial therapy was continued for a total duration of 21 days as per the recommended standard of care for post-neurosurgical meningitis. The child improved neurologically and was later transferred to the neuro-rehabilitation unit for further care.

Informed consent was obtained from the patient’s guardian for publication of this case report and accompanying images.

## Discussion

The *Acinetobacter* genus includes more than 50 environmental species, but human disease is primarily due to *A. baumannii*, with less frequent involvement of *A. calcoaceticus* and *A. lwoffii*. Infections are encountered predominantly in intensive care and post-neurosurgical settings, where pneumonia and bloodstream infections are most common, while urinary tract infection, post-neurosurgical meningitis, wound infection, and osteomyelitis occur less frequently. The accumulation of intrinsic and acquired resistance mechanisms in CRAB markedly limits available therapeutic options [3].

Current management strategies for CRAB infections often include polymyxins, tigecycline, or sulbactam-based regimens [4]. Among these, high-dose ampicillin-sulbactam has gained renewed interest because sulbactam itself possesses intrinsic bactericidal activity against* A. baumannii* [5]. Observational studies suggest that combining colistin with high-dose ampicillin-sulbactam is associated with improved clinical outcomes and lower mortality compared with colistin monotherapy [3,4].

In pediatric patients, ampicillin-sulbactam dosing typically ranges up to 300 mg/kg/day administered intravenously in divided doses every six hours, and this regimen has demonstrated good clinical efficacy and tolerability [5,6]. The favorable clinical response observed in this case supports prior findings that ampicillin-sulbactam, when used in combination therapy, may offer a valuable alternative to colistin or polymyxin-based regimens, especially in cases of multidrug-resistant *A. baumannii* infection.

A structured, evidence-based treatment approach for CRAB infections has been described in the literature, emphasizing individualized therapy guided by antimicrobial susceptibility results and infection site [7]. Our case further supports the growing evidence that sulbactam-containing regimens can be a safe and effective component of therapy for CRAB meningitis, particularly in the pediatric population.

## Conclusions

Ampicillin-sulbactam was successfully used in this case to treat post-neurosurgical meningitis caused by CRAB. The favorable clinical and microbiological response highlights its potential role as part of combination therapy in managing multidrug-resistant infections, particularly when alternative options such as polymyxins carry higher toxicity risks.

This case adds to the limited but growing evidence supporting the efficacy and safety of ampicillin-sulbactam in pediatric CRAB infections. Further case reports and prospective studies are needed to establish standardized dosing regimens and to evaluate long-term outcomes in both adult and pediatric populations.
